# Evaluating the appropriateness of chemotherapy in a low‐resource cancer centre in sub‐Saharan Africa

**DOI:** 10.1002/cam4.2672

**Published:** 2019-11-13

**Authors:** Patrizia Serra, Deogratias M. Katabalo, Nestory Masalu, Dino Amadori, Salustia Bugingo, Flavia Foca, Sara Bravaccini, Caterina Donati, Lauro Bucchi, Carla Masini

**Affiliations:** ^1^ Biostatistics and Clinical Trials Unit Istituto Scientifico Romagnolo per lo Studio e la Cura dei Tumori (IRST) IRCCS Meldola Italy; ^2^ Oncology Department Bugando Medical Centre Mwanza Tanzania; ^3^ Istituto Oncologico Romagnolo Forlì Italy; ^4^ Gerobiomics Unit, Bioscience Laboratory Istituto Scientifico Romagnolo per lo Studio e la Cura dei Tumori (IRST) IRCCS Meldola Italy; ^5^ Oncology Pharmacy Unit Istituto Scientifico Romagnolo per lo Studio e la Cura dei Tumori (IRST) IRCCS Meldola Italy; ^6^ Romagna Cancer Registry Istituto Scientifico Romagnolo per lo Studio e la Cura dei Tumori (IRST) IRCCS Meldola Italy

**Keywords:** Africa, appropriateness, cancer, chemotherapy

## Abstract

**Background:**

To evaluate the appropriateness of chemotherapy use at the Oncology Department of the Bugando Medical Centre of Mwanza, Tanzania.

**Methods:**

The study was an observational prevalence‐based study designed to evaluate a single‐chemotherapy cycle during a defined time period for a cross‐section of patients at varying stages of their clinical history. The sample included 103 consecutive subjects who were treated during January‐March 2017 and had at least one previous cycle. Chemotherapy treatment omissions, cycle delays, and dose reductions and their causes were recorded using a standard form that included demographic, anthropometric, and clinical items. The data were analyzed descriptively.

**Results:**

There were 59 males (57.3%) and 44 females (42.7%). Ninety‐four patients were aged ≥18 years. Considering cancer type/site, there were 23 distinct groups of patients. The recorded number of drugs in the chemotherapy regimens varied between one and five. The median cycle number was three (range: 2‐11). Sixty‐eight (66.0%) patients were treated in a standard fashion. For the remaining, cycle delay and dose reduction were the most common cause for nonstandard treatment. Hematologic toxicity was responsible for the greater part of cycle delays, whereas dose reductions were accounted for by a larger spectrum of causes. Overall, toxicity explained 21/35 (60.0%) patients receiving nonstandard treatment. The distribution of toxic events was skewed toward grade 1 and grade 2.

**Conclusions:**

The observed level of appropriateness of chemotherapy was encouraging. The proportion of patients experiencing severe toxic effects was lower than expected.

## Graphical abstract

The study aims to evaluate the appropriateness of chemotherapy use at the Oncology Department of the Bugando Medical Centre of Mwanza, Tanzania. The study was an observational prevalence‐based study designed to evaluate a single‐chemotherapy cycle during a defined time period for a cross‐section of patients at varying stages of their clinical history. The observed level of appropriateness of chemotherapy was encouraging. The proportion of patients experiencing severe toxic effects was lower than expected.

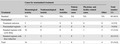

## 1 INTRODUCTION

In most sub‐Saharan African countries, where cancer rates are increasing,^1^, ^2^ comprehensive programmes for the control of the disease are lacking, and diagnostic and treatment capacity is insufficient.^3^, ^4^ The major obstacles to be removed in order to improve patients' access to effective treatment include little infrastructure, high cost of oncological care, and shortage of qualified surgeons, medical oncologists, pathologists, radiation oncologists, and other health‐care workers.^5^, ^6^, ^7^

Access to cancer chemotherapy is further limited by many specific challenges. Nonspecialist physicians have difficulties to correctly prescribe, prepare, and administer the drugs and to monitor their side effects. In addition, almost all types of cancer are at an advanced stage at presentation.^8^ This would require more toxic and more expensive therapy, but patients rarely have insurance. In general, socioeconomic deprivation results in patients failing to complete therapy. The use of generic drugs, too, is widespread. When drugs are available and affordable, there are often problems with protocol adherence by the physician or the patient, or because of excessive toxicity.^9^ Delays in referral for adjuvant treatment, too, remain a major issue, that can be attributed to cultural beliefs that regard surgery as a taboo and to denial (especially after complete clinical response).^6^ For all of these reasons, even the limited resources available are often inefficiently used.^9^

Until the end of 2018, the costs of drugs were charged to the patients, except children aged ≤5 years and adults aged ≥65 years. However, two strategic objectives of the Tanzania National Cancer Control Strategy (NCCS) for the years 2013‐2022 are (#3.3) to provide access to best available drugs and treatment procedures and (#9.1) to reduce inequalities in respect to access to cancer care.^10^ This includes the provision of pharmaceutical care to all uninsured cancer patients irrespective of age. The implementation of the NCCS is underway.

In this article, we report a study on the appropriateness of chemotherapy use at the Oncology Department of the Bugando Medical Centre (BMC) of Mwanza, Tanzania. Its rationale was that the gradual implementation of the NCCS makes it more and more urgent the need to correctly prescribe, prepare, and administer chemotherapy drugs.

In detail, the objectives of the study were: (a) to determine the proportion of patients admitted in a defined time period who were treated with standard chemotherapy regimens at the standard doses and at the scheduled times; and (b) to classify the remaining ones according to the type of mistreatment (treatment omission, nonstandard regimen, standard regimen with cycle delay, standard regimen with dose reduction) and according to the cause of mistreatment (hematological toxicity, nonhematological toxicity, patient‐related cause, physician‐ or facility‐related cause). A secondary objective of the study was to test the tool developed for assessing toxicity.

## 2 METHODS

### 2.1 Setting

The BMC of Mwanza is the largest referral hospital in the Lake zone of north‐western Tanzania. The catchment area includes eight districts with a population, according to the Tanzania Bureau of Statistics, of approximately 14 millions.

The Medical Oncology Unit (subsequently renamed Oncology Department) was established in 2009 as a part of a collaborative multi‐step initiative, termed the Mwanza Cancer Project, between a pool of Italian cancer research centers, cancer volunteer associations and scientific societies, Tanzanian political and health authorities, and health‐care professionals at the BMC. The project was aimed at creating a comprehensive cancer centre. More details can be found elsewhere.^4^, ^8^, ^11^, ^12^

Currently, the annual number of admissions to the Oncology Department is about 7000, the greater part of which is on an outpatient basis. Chemotherapy treatments are given in a dedicated ambulatory with 27 treatment beds that is open 3 days a week for 8 hours daily. Chemotherapy treatment is based on conventional cancer drugs. Targeted drugs are not the part of the standard‐of‐care therapy in Tanzania. The ultimate decision on treatment rests with the Director of the Department. There is no institutional supervision on the type of drugs and regimens being used. The pharmaceutical department of the BMC is directed by a pharmacist and provides consultations for the physicians who request assistance.

### 2.2 Training

The Mwanza Cancer Project includes, among others, regular practical/theoretical training programmes held both on‐site and in Italy. Physicians, pharmacists, nurses, and pharmaceutical technicians participate in residential courses in prescription, preparation, and administration of chemotherapy drugs in Italy, with durations varying between 1 month for the pharmacists and 12 months for the physicians, followed by refreshing courses at the BMC.

In 2013, an electronic clinical case record system was installed at the Oncology Department which provides information on standard treatments for defined conditions and permits to calculate the dosage of drugs and the duration of treatment. This software is described elsewhere.^4^

### 2.3 Design

The study was conceived as a retrospective observational prevalence‐based study designed to measure the appropriateness of chemotherapy treatment in a single cycle during a defined time period for a cross‐section of patients at varying stages of their clinical history. It was targeted at a sample of 103 consecutive patients with onco‐hematologic malignancies who met the following eligibility criteria: having been treated with intravenous drugs; having been treated during January, February, and March 2017; and having had at least one previous cycle, and thus being at the risk of toxicity.

### 2.4 Definitions

Nonstandard chemotherapy regimen was defined as a regimen other than those listed, for each tumor type, in Table 1. The regimens in Table 1, indicated by the Director of the Oncology Department, are based on scientific evidence (though with limited exceptions caused by external factors).

**Table 1:** Number of patients by cancer type and standard chemotherapy regimen (n = 103)

Cancer type	ICD‐10	Standard regimen	No.
Bladder carcinoma	C67	CDDP (Cisplatin)	1
		CDDP G (Cisplatin‐Gemcitabine)	1
		CG (Cisplatin/Carboplatin‐Gemcitabine)	2
Brain tumor	C70‐C72	VEC (Vincristine‐Etoposide‐Cyclophosphamide)	1
Breast cancer	C50	AC (Doxorubicin‐Cyclophosphamide)	1
		CAF (Cyclophosphamide‐Doxorubicine/Adriamycin‐5‐Fluorouracyl)	5
		CMF (Cyclophosphamide‐Methotrexate‐5‐Fluorouracyl)	2
		CP (Carboplatin‐Paclitaxel)	1
		Capecitabine	1
		DGC (Docetaxel‐Gemcitabine‐Carboplatin)	1
		Docetaxel	1
		Gemcitabine	2
		PA (Paclitaxel‐Doxorubicin)	3
		PAC (Paclitaxel‐Adriamycin‐Cyclophosphamide)	1
		Paclitaxel	1
Cervical carcinoma	C53	CF (Cisplatin‐5‐Fluorouracyl)	2
		Carboplatin	1
		Paclitaxel	1
Choriocarcinoma	C58	CDDP E (Cisplatin‐Etoposide)	1
		EMACO (Etoposide‐Methotrexate‐Dactinomycin‐Vincristine‐Cyclophosphamide)	1
		EMO (Methotrexate‐Vincristine‐Etoposide)	1
		GED (Gemcitabine‐Etoposide‐Dacarbazine)	1
		MEDF (Methotrexate‐Etoposide‐Dactinomycin‐Folic Acid)	1
		Methotrexate	2
Endometrial carcinoma	C54	CF (Cisplatin‐5‐Fluorouracyl)	1
Chronic lymphocytic** **leukemia	C91	Chlorambucil	1
Esophageal carcinoma	C15	CF (Cisplatin‐5‐Fluorouracyl)	3
		Docetaxel	1
Orbital squamous cell carcinoma	C44	CF (Cisplatin‐5‐Fluorouracyl)	3
Gastric cancer	C16	XELOX (Capecitabine‐Oxaliplatin)	1
Hepatocarcinoma	C22	Doxorubicin	7
		Doxorubicin‐5‐Fluorouracyl	1
		Doxorubicin liposomal	2
Hodgkin lymphoma	C81	ABVD (Adriamycin‐Bleomycin‐Vinblastine‐Dacarbazine)	6
Kaposi sarcoma	C46	ABV (Doxorubicin‐Bleomycin‐Vincristine)	2
		AV (Doxorubicin‐Vincristine)	8
		DC (Doxorubicin‐Cyclophosphamide)	1
		Docetaxel	3
		DVC (Doxorubicin‐Cisplatin‐Vincristine)	1
		Doxorubicin liposomal	3
		Paclitaxel	3
		PV (Paclitaxel‐Vincristine)	1
Laryngeal carcinoma	C32	DCF (Docetaxel‐Cisplatin‐5‐Fluorouracyl)	1
Lung	C34	Docetaxel	1
		GC (Gemcitabine‐Cisplatin)	1
Non‐Hodgkin lymphoma	C82‐C85, C96	CHOP (Cyclophosphamide‐Doxorubicin‐Vincristine‐Prednisone)	7
		DC (Doxorubicin‐Cisplatin)	1
		MCV (Methotrexate‐Cyclophosphamide‐Vincristine)	1
Neuroendocrine	C75	EC (Etoposide‐Cisplatin)	1
Ovarian cancer	C56	CP (Carboplatin‐Paclitaxel)	2
Pancreatic cancer	C25	Gemox (Gemcitabine‐Oxaliplatin‐5‐Fluorouracyl)	1
Rectal cancer	C19‐C21	Xelox (Oxaliplatin‐Capecitabine)	1
Rectal squamous cell carcinoma	C19‐C21	5‐Fluorouracyl‐Carboplatin	1
Retinoblastoma	C69	MCVE‐CDDP (Methotrexate‐Cyclophosphamide‐Vincristine‐Etoposide + Cisplatin)	1
Stromal stem cells carcinoma	C49	CF (Cisplatin‐5‐Fluorouracyl)	1

Treatment omission was defined as no further treatment or a cycle delay equal to, or greater than, the scheduled interval between chemotherapy cycles. Cycle delay was defined as a cycle administered within 1 week after the scheduled date, and within 2 days for weekly regimens. Dose reduction was defined as a decrease in chemotherapy dose ≥20% relative to the standard.

Patient‐related causes of mistreatment were considered to include: nonadherence to prescribed chemotherapy treatment, denial of illness, frustration, social stigma, economic problems, and cultural beliefs. Physician‐ and facility‐related causes were considered to include the late delivery of clinical chemistry reports, prescription errors, partial or total unavailability of drugs, and overbooking of patients.

### 2.5 Data collection and management

A standard record form was developed in two steps. The final version was validated in a study on the effects of pharmacist participation on therapeutic outcomes, including verified dosage calculations, chemotherapy administration adherence, and dose documentation.^13^

The form was completed for each patient by the treating physician (see form, File S1). Information was retrieved from the paper‐based patient clinical notes and the electronic records (for blood clinical‐chemical parameters and radiology reports). The form included demographic, anthropometric, and clinical items. Regarding the chemotherapy regimen, the date of administration, the name of drug(s), the number of cycle, the scheduled dose, and the dose administered were recorded. Performance status was classified according to the Eastern Cooperative Oncology Group (ECOG). Information regarding toxicity was recorded according to the Common Terminology Criteria for Adverse Events Version 5.0. At the end of recruitment, an external check of data by one of the Italian partners was done on‐site. Another check of data quality, focusing on records with apparent medical inconsistencies, was carried out by the Director of the Department (NM). The data were entered in a Microsoft Excel worksheet and analyzed descriptively in Italy. Categorical variables were presented as numbers and percentages. Continuous variables were presented using median and range. Data management and descriptive analyses were carried out with STATA/MP 15.0 for Windows (StataCorp LLP).

## 3 RESULTS

Table 2 shows the general characteristics of patients. Both the patient age range and the spectrum of diseases were broad, reflecting the mixture of pediatric and adult patients with solid tumors and hematologic malignancies. The median age, however, was as low as 41 years. The proportion of patients with a good ECOG performance status, that is, 0‐2, was >90%. The high prevalence of patients presenting with Kaposi sarcoma was expected, reflecting the alarming rise in the prevalence of human immunodeficiency virus in the region. The high prevalence of patients with hepatocarcinoma indicated that sub‐Saharan Africa is an area with endemic hepatitis B and hepatitis C virus infection and with excessive exposition to aflatoxin B1. Another correlate of high rates of hepatocarcinoma was the high male to female ratio, that is, 4.1 (not shown).

**Table 2:** General characteristics of patients (n = 103)

Characteristic	No. (%)^a^
Patient age	
Median	41
Range	6‐76
Pediatric^b^	9 (8.7)
Adult^c^	94 (91.3)
Gender	
Male	59 (57.3)
Female	44 (42.7)
Performance status^d^	
0	12 (11.7)
1	45 (43.7)
2	37 (35.9)
3	9 (8.7)
Site of disease	
Kaposi sarcoma	22 (21.4)
Hemolymphoproliferative tumors	
Non‐Hodgkin lymphoma	9 (8.8)
Hodgkin lymphoma	6 (5.8)
Chronic lymphocytic leukemia	1 (1.0)
Choriocarcinoma	7 (6.8)
Epithelial solid tumors	
Breast	19 (18.4)
Hepatocarcinoma	10 (9.7)
Larynx, gastric, or esophageal cancer	6 (5.8)
Other site of disease	23 (22.3)

a Except for median age and age range (years).

b <17 years.

c ≥18 years.

d Classified according to the Eastern Cooperative Oncology Group.

The number of patients by cancer type/site and standard chemotherapy regimen is shown in the column at right in Table 1. With respect to cancer type/site, we identified 23 distinct groups of patients.

The recorded number of drugs in the regimens was 1 for 30 patients (29.1%), 2 for 39 (37.9%), 3 for 18 (17.5%), 4 for 14 (13.6%), and 5 for two (1.9%). The median cycle number evaluated in the study was three, with a range of 2 to 11.

Table 3 shows the proportion of patients receiving standard regimens and the distribution of those treated in a nonstandard way by type and cause of mistreatment. Sixty‐eight (66%) patients received a standard regimen. Cycle delay and dose reduction were the most common causes for patients receiving nonstandard regimens. Hematologic toxicity, and not patient‐related causes nor physician‐ and facility‐related causes, was responsible for the greater part of cycle delays, whereas dose reductions were accounted for by a larger spectrum of causes. Overall, toxicity explained 21/35 (60.0%) patients receiving nonstandard treatment, whereas factors relating to the patient, the physician, and the facility accounted for as few as 10 (28.6%) of them.

**Table 3:** Number of patients with nonstandard treatment by cause (n = 103)

Treatment	Cause for nonstandard treatment						Total number (%)
	Hematological toxicity	Nonhematological toxicity	Both toxicities	Patient‐related cause	Physician‐ and facility‐related cause	Other	
Standard	NA	NA	NA	NA	NA	NA	68 (66.0)
Nonstandard							
Treatment omission	5	1	0	0	0	0	6 (5.9)
Nonstandard regimen	0	0	0	0	0	0	0 (0.0)
Standard regimen with cycle delay	10	0	0	0	1	2	13 (12.6)
Standard regimen with dose reduction	1	2	2	8	1	2	16 (15.5)
Total	16	3	2	8	2	4	35 (34.0)

Abbreviation: NA, not applicable.

Table 4 shows the distribution of the toxic events observed according to their grade. Seldom patients developed the most severe toxicity (grade 4). The distribution was skewed toward grade 1 and grade 2.

**Table 4:** Number (and percent distribution) of reported toxic events by toxicity grade (n = 103)

Toxic event	Toxicity grade^a^			
	1	2	3	4
Anemia	41 (39.8)	17 (16.5)	5 (4.9)	3 (2.9)
Neutropenia	46 (44.7)	13 (12.6)	9 (8.7)	4 (3.9)
Febrile neutropenia	2 (1.9)	0 (0.0)	15 (14.6)	0 (0.0)
Hyperuricemia	23 (22.3)	1 (1.0)	3 (2.9)	0 (0.0)
Nausea	34 (33.0)	33 (32.0)	6 (5.8)	0 (0.0)
Vomiting	36 (35.0)	23 (22.3)	7 (6.8)	0 (0.0)
Mucositis	22 (21.4)	10 (9.7)	3 (2.9)	1 (1.0)
Diarrhea	18 (17.5)	3 (2.9)	4 (3.9)	1 (1.0)
Constipation	12 (11.7)	0 (0.0)	1 (1.0)	0 (0.0)
Fever	15 (14.6)	2 (1.9)	1 (1.0)	0 (0.0)
Palmar syndrome	29 (28.2)	3 (2.9)	1 (1.0)	0 (0.0)
Fatigue	52 (50.5)	19 (18.4)	3 (2.9)	0 (0.0)
Anorexia	33 (32.0)	43 (41.7)	3 (2.9)	0 (0.0)
Dyspnea	37 (35.9)	4 (3.9)	0 (0.0)	1 (1.0)
Rush	12 (11.7)	3 (2.9)	2 (1.9)	0 (0.0)
Peripheral neuropathy	20 (19.4)	7 (6.8)	2 (1.9)	0 (0.0)
Pain	32 (31.1)	10 (9.7)	1 (1.0)	0 (0.0)

a Toxicity was classified according to the Common Terminology Criteria for Adverse Events Version 5.0.

## 4 DISCUSSION

The design of this research requires a clarification. In view of the implementation of the NCCS, we were interested in evaluating both the medical and the institutional/environmental determinants of inappropriate chemotherapy use. Regarding the latter, the NCCS is gradually creating a new scenario. After the conclusion of the study, for example, we have experienced problems with drug delivery (late delivery, under‐delivery, lack of delivery, low shelf life) by the Medical Store Department of Dar es Salaam, a semiautonomous, public, non‐for‐profit structure that is responsible for procurement and distribution of drugs at the national level.^14^ It must be noted that the validity of our observations regarding the medical causes of nonstandard treatment is independent of these changing conditions.

Overall, the observed level of appropriateness of chemotherapy at the Oncology Department of the BMC was encouraging. Two‐thirds of patients received standard chemotherapy regimens. Also, the greater part of cycle delays were accounted for by hematologic toxicity—and not patient‐related causes nor physician‐ and facility‐related causes. More specifically, the proportion of nonstandard treatments due to hematologic toxicity was comparable with that commonly observed in case series from the developed countries. This indicates that substantial errors in therapeutic decisions did not occur. Our findings should be considered taking into account that toxicity was assessed using the Common Terminology Criteria for Adverse Events Version 5.0 and, thus, in a standard way.

A related observation of interest was that the large majority of regimens were correctly identified as standard regimens by the treating physician, despite the fact that the diseases included in the sample were highly heterogeneous.

The proportion of patients experiencing severe toxic effects was lower than is commonly seen among Caucasian patients. Considering the good proportion of patients treated with full‐dose standard regimens, a wider range of toxic events was expected. As the consistency and quality of data were checked on‐site by one of the Italian partners, there remain only two (not mutually exclusive) hypotheses to explain the low proportion of grade 4 toxicities.

First, we cannot exclude that the data suffer from some degree of underreporting of subjective symptoms by patients, which may be due to embarrassment about them, fear, declining motivation over time, and stigma attached to cancer.^15^

Second, our data are compatible with the hypothesis that sub‐Saharan cancer patients may have a different susceptibility to toxic events compared with Caucasian patients. To confirm this, ad hoc studies are needed. The identification of individuals at low (and high) risk of drug resistance or toxicity involves the exploration of interindividual variations at the DNA sequence level resulting in altered expression levels and/or activities of the encoded proteins.^16^ The effect of several polymorphisms on the pharmacokinetic behavior of chemotherapy drugs has been investigated in Asian^17^ and Latin American^18^ populations.

In this study, chemotherapy dose reduction was defined according to standard criteria. In sub‐Saharan Africa, however, this question must be viewed from a different standpoint than in the developed countries. In this region, using less drugs and implementing shorter‐duration treatment protocols must be viewed as potential means to reduce the cost of chemotherapy. However, this approach warrants more research relevant to local patient populations and treatment facilities.^9^ From this perspective, developing a sensitive and suitable tool to assess toxicity during chemotherapy has been an important step toward enabling the BMC to give a contribution to this research effort.

There are two major weaknesses in this study. First, the sample size was limited by objective difficulties in collecting the data for a sustained period of time. Second, we were unable to compare the sample of patients with data on cancer prevalence in the area—that are still lacking. Assuming that patients were regularly recruited, the composition of the sample depended on the interaction between disease‐specific prevalence and other factors, in particular the planned number and frequency of cycles within a course of treatment. In any case, the broad patient age range and the diverse spectrum of diseases suggested that the sample was fairly representative of the whole population usually attending the BMC.

## 5 CONCLUSION

In summary, two‐thirds of patients were treated in a standard fashion. Cycle delays and dose reductions were the most common causes for patients receiving nonstandard regimens. Hematologic toxicity was responsible for the greater part of delays. Problems accounting for dose reduction were more heterogeneous. The proportion of patients experiencing severe toxic effects was lower than expected.

## CONFLICT OF INTEREST

None declared.

## AUTHOR CONTRIBUTIONS

Patrizia Serra, Deogratias M. Katabalo, Nestory Masalu, Dino Amadori, and Carla Masini conceived the study. Sara Bravaccini supervised the data collection. Flavia Foca analyzed the data. Salustia Bugingo, Caterina Donati, and Lauro Bucchi drafted the manuscript. All authors read and approved the present version of the manuscript for submission.

## ETHICS APPROVAL AND CONSENT TO PARTICIPATE

The study protocol was approved by the Ethical Committee at the Istituto Scientifico Romagnolo per lo Studio e la Cura dei Tumori (IRST) IRCCS, Meldola, Italy (ID: IRST100.37); was also conducted in accordance with the Declaration of Helsinki and all patients signed the informed consent.

## Data Availability

The datasets generated and/or analyzed during the current study are available from the corresponding author on reasonable request.
