# Supplementary material for: Evaluating the appropriateness of chemotherapy in a low‐resource cancer centre in sub‐Saharan Africa
Source: Cancer Med. 2019 Nov 13;9(1):133–40. doi: 10.1002/cam4.2672 (PMC6943087; doi:10.1002/cam4.2672)
Supplement: Supplementary file 1 [file CAM4-9-133-s001.pdf]

TREATMENT

Name: ________________________ Surname: _______________________ Date of Birth ___________

Weight Kg: __________ Height cm: ___________ PS______________ BSA_____________________

Site of disease______________________

Subject N. : _ _ _

Age: _ _

Sex: ☐ 1. M ☐ 2. F

Cycle N. _ _

Treatment:

Schedule:

Date of last administration _ _/_ _/_ _ _ _

Date of current administration_ _/_ _/_ _ _ _

DRUG	Treatment Omitted*	Treatment Delayed*	Dose Reduction*	Total dose Administered	Dose Administered
1.				mg	mg/m^2
2.				mg	mg/m^2
3.				mg	mg/m^2
4.				mg	mg/m^2

*0 = NO

1 = Yes, hematological toxicity

2 = Yes, NON hematological toxicity

3 = Yes, Both

4 = Related to patient

5 = Related to center

6 = Other, specify ____

TOXICITY

Adverse Event			GRADE				
		0	1	2	3	4	5
**Anemia**		None	Hemoglobin (Hgb) <LLN - 10.0 g/dL; <LLN - 6.2 mmol/L; <LLN - 100 g/L	Hgb <10.0 - 8.0 g/dL; <6.2 - 4.9 mmol/L; <100 - 80g/L	Hgb <8.0 - 6.5 g/dL; <4.9 - 4.0 mmol/L; <80 - 65 g/L; transfusion indicated	Life-threatening consequences; urgent intervention indicated	Death
**Neutropenia**			<LLN - 1500/mm3; <LLN - 1.5 x 10e9 /L	<1500 - 1000/mm3; <1.5 - 1.0 x 10e9 /L	<1000 - 500/mm3; <1.0 - 0.5 x 10e9 /L	00/mm3; <0.5 x 10e9 /L	
**Febrile neutropenia**		None	-	-	Present	Life-threatening consequences; urgent intervention indicated	Death
**Hyperuricemia**		None	>ULN - 10 mg/dL; <=0.59 mmol/L without physiologic consequences	-	>ULN - 10 mg/dL; <=0.59 mmol/L with physiologic consequences	>10 mg/dL; >0.59 mmol/L; life-threatening consequences	Death
**Nausea**		None	Loss of appetite without alteration in eating habits	Oral intake decreased without significant weight loss, dehydration or malnutrition	Inadequate oral caloric or fluid intake; tube feeding, TPN, or hospitalization indicated	-	-
**Vomiting**		None	1 - 2 episodes (separated by 5 minutes) in 24 hrs	3 - 5 episodes (separated by 5 minutes) in 24 hrs	>=6 episodes (separated by 5 minutes) in 24 hrs; tube feeding, TPN or hospitalization indicated	Life-threatening consequences; urgent intervention indicated	Death
**Mucositis/Stomatitis**		None	Asymptomatic or mild symptoms; intervention not indicated	Moderate pain; not interfering with oral intake; modified diet indicated	Severe pain; interfering with oral intake	Life-threatening consequences; urgent intervention indicated	Death
**Diarrhea**		None	Increase of <4 stools per day over baseline; mild increase in ostomy output compared to baseline	Increase of 4 - 6 stools per day over baseline; moderate increase in ostomy output compared to baseline	Increase of >=7 stools per day over baseline; incontinence; hospitalization indicated; severe increase in ostomy output compared to baseline; limiting self care ADL	Life-threatening consequences; urgent intervention indicated	Death
**Constipation**		None	Occasional or intermittent symptoms; occasional use of stool softeners, laxatives, dietary modification, or enema	Persistent symptoms with regular use of laxatives or enemas; limiting instrumental ADL	Obstipation with manual evacuation indicated; limiting self care ADL	Life-threatening consequences; urgent intervention indicated	Death
**Fever (NOT reciving chemot**		None	38.0 - 39.0 degrees C (100.4 - 102.2 degrees F)	>39.0 - 40.0 degrees C (102.3 - 104.0 degrees F)	>40.0 degrees C (>104.0 degrees F) for <=24 hrs	>40.0 degrees C (>104.0 degrees F) for >24 hrs	Death
**Palmar-plantar erythrodysesthesia syndrome**		None	Minimal skin changes or dermatitis (e.g., erythema, edema, or hyperkeratosis) without pain	Skin changes (e.g., peeling, blisters, bleeding, edema, or hyperkeratosis) with pain; limiting instrumental ADL	Severe skin changes (e.g., peeling, blisters, bleeding, edema, or hyperkeratosis) with pain; limiting self care ADL	-	Death
**Fatigue**		None	Fatigue relieved by rest	Fatigue not relieved by rest; limiting instrumental ADL	Fatigue not relieved by rest, limiting self care ADL	-	Death
**Anorexia**		None	Loss of appetite without alteration in eating habits	Oral intake altered without significant weight loss or malnutrition; oral nutritional supplements indicated	Associated with significant weight loss or malnutrition (e.g., inadequate oral caloric and/or fluid intake); tube feeding or TPN indicated	Life-threatening consequences; urgent intervention indicated	Death
**Dyspnea**		None	Shortness of breath with moderate exertion	Shortness of breath with minimal exertion; limiting instrumental ADL	Shortness of breath at rest; limiting self care ADL	Life-threatening consequences; urgent intervention indicated	Death
**Rash maculo-papular**		None	Macules/papules covering <10% BSA with or without symptoms (e.g., pruritus, burning, tightness)	Macules/papules covering 10 - 30% BSA with or without symptoms (e.g., pruritus, burning, tightness); limiting instrumental ADL	Macules/papules covering >30% BSA with or without associated symptoms; limiting self care ADL	-	Death
**Peripheral sensory neuropathy**		None	Asymptomatic; loss of deep tendon reflexes or paresthesia	Moderate symptoms; limiting instrumental ADL	Severe symptoms; limiting self care ADL	Life-threatening consequences; urgent intervention indicated	Death
**Tumor pain**		None	Mild pain	Moderate pain; limiting instrumental ADL	Severe pain; limiting self care ADL	- H20	-
