# Supplementary material for: Evaluating the appropriateness of chemotherapy in a low‐resource cancer centre in sub‐Saharan Africa
Source: Cancer Med. 2019 Nov 13;9(1):133–40. doi: 10.1002/cam4.2672 (PMC6943087; doi:10.1002/cam4.2672)
Supplement: Supplementary file 2 [file CAM4-9-133-s002.docx]

**Form legend:** The standard record form developed for the study. The form included demographic, anthropometric and clinical items. Performance status was classified according to the Eastern Cooperative Oncology Group. Information regarding toxicity was recorded according to the Common Terminology Criteria for Adverse Events Version 5.0.
